# One Anastomosis/Mini-Gastric Bypass (OAGB/MGB) as Revisional Surgery Following Primary Restrictive Bariatric Procedures: a Systematic Review and Meta-Analysis

**DOI:** 10.1007/s11695-020-05079-x

**Published:** 2020-10-28

**Authors:** Mohammad Kermansaravi, Shahab Shahabi Shahmiri, Amir Hossein DavarpanahJazi, Rohollah Valizadeh, Giovanna Berardi, Antonio Vitiello, Mario Musella, Miguel Carbajo

**Affiliations:** 1 Department of Surgery, Minimally Invasive Surgery Research Center, Division of Minimally Invasive and Bariatric Surgery, Rasool-e Akram Hospital, Iran University of Medical Sciences, Tehran, Iran; 2 Minimally Invasive Surgery Research Center, Isfahan University of Medical Sciences, Isfahan, Iran; 3 Department of Epidemiology, Student Research Committee, School of Public Health, Iran University of Medical science, Tehran, Iran; 4 Advanced Biomedical Sciences Department, “Federico II” University, Naples, Italy; 5 Centre of Excellence for the Study and Treatment of Obesity and Diabetes, Valladolid, Spain

**Keywords:** One anastomosis gastric bypass (OAGB/MGB), Gastric bypass, Weight regain, Weight loss, Conversion

## Abstract

One anastomosis gastric bypass (OAGB/MGB) has gained popularity in the past decade. International databases were searched for articles published by September 10, 2020, on OAGB/MGB as a revisional procedure after restrictive procedures. Twenty-six studies examining a total of 1771 patients were included. The mean initial BMI was 45.70 kg/m^2^, which decreased to 31.52, 31.40, and 30.54 kg/m^2^ at 1, 3, and 5-year follow-ups, respectively. Remission of type-2 diabetes mellitus (T2DM) following OAGB/MGB at 1-, 3-, and 5-year follow-up was 65.16 ± 24.43, 65.37 ± 36.07, and 78.10 ± 14.19%, respectively. Remission/improvement rate from gastroesophageal reflux disease (GERD). Also, 7.4% of the patients developed de novo GERD following OAGB/MGB. Leakage was the most common major complication. OAGB/MGB appears to be feasible and effective as a revisional procedure after failed restrictive bariatric procedures.

**Supplementary Information:**

The online version contains supplementary material available at 10.1007/s11695-020-05079-x.

## Introduction

In last years, bariatric surgery has proven to be the most effective treatment for morbid obesity and obesity-related diseases, providing long-standing effects and presenting a very low complication rate [1–3]. One anastomosis gastric bypass was firstly introduced by Rutledge as mini-gastric bypass (MGB) in 2001 [4] and subsequently modified as OAGB by Carbajo in 2005 [5]. It is currently an accepted bariatric procedure named as OAGB/MGB by IFSO [6], which has gained increasing popularity among bariatric surgeons worldwide [7]; efficacy and safety of primary OAGB/MGB have been reported in many different papers [8–12]. Due to the growing request for revisional bariatric surgery that recorded a steep increase in last years, rising from 6 to 13.6% of all bariatric procedures, a number of articles about conversional OAGB/MGB following primary restrictive procedures have been already published. Current revisional surgery rates are in fact reported to range from 9.8% for laparoscopic sleeve gastrectomy, to 26% for laparoscopic adjustable gastric banding [13]. Although a systematic review of the studies reporting results and complications of conversional OAGB/MGB has been very recently released [14], a study offering an analytic approach to conversional OAGB/MGB following laparoscopic adjustable gastric banding (LAGB), laparoscopic sleeve gastrectomy (LSG), and vertical banded gastroplasty (VBG) is still lacking. Aim of this study is therefore to define through a meta-analysis the role of OAGB/MGB as a conversional procedure after failed restrictive procedures, such as LAGB, LSG, and VBG.

## Methods

A literature search was carried out based on the Preferred Reporting Items for Systematic Review and Meta-Analyses (PRISMA) guidelines [15] (see supplementary material). PubMed, Cochrane, and Scopus were consulted for articles published by September 10, 2020, on OAGB/MGB as a revisional procedure following restrictive procedures. The keywords searched were “One anastomosis gastric bypass,” “OAGB,” “Single-anastomosis gastric bypass,” “Weight regain,” “Weight loss,” “Conversion,” “Mini gastric bypass,” “MGB,” “Failure,” “Redo,” “Revisional surgery,” “Conversional surgery,” “omega loop gastric bypass,” or “loop gastric bypass,” “revisional bariatric surgery,” “secondary bariatric surgery,” “revisional sleeve gastrectomy,” “band to bypass,” “revision to bypass,” or a combination of them in the titles or abstracts. The search strategy for can be found in the supplementary files. Two of the authors independently assessed the eligibility of the papers according to the PRISMA guidelines. The references of the articles were manually reviewed for additional relevant papers. The duplicate studies were removed.

### Statistical Analysis

The effect of revisional surgery on body mass index (BMI) was assessed using the standardized mean difference (SMD), also known as Cohen’s D. The SMD was calculated by using the mean difference and standard deviations (SD) before and after the surgery based on the SMD formula (SMD = mean difference in the intervention group − mean difference in the placebo group/pooled SD), and the pooled SD was calculated as √ [(SD in the intervention group) 2 + (SD in the placebo group) 2/2]. Q-test and I^2^ were used to assess the heterogeneity among the studies. The random-effects model was used for the continuous outcome under study. Also, a random or fixed-effects meta-analysis was applied for estimating the main index, which was the pooled SMD, at 95% confidence interval. A forest plot was used to present the pooled SMD. Publication bias was assessed using Begg’s tests. The analysis was performed using Stats version 13.

### Data Extraction

Data on the included articles (author’s name, year of publication, interval to revision, sample size, type of primary surgery, and the outcomes and results of each article) were retrieved by two independent investigators. The differences observed in this process were corrected by a third investigator independent from the other two.

The quality of the selected studies was checked by a quality assessment tool for before-after (pre-post) studies with no control groups [16].

## Results

**Fig. 1:**
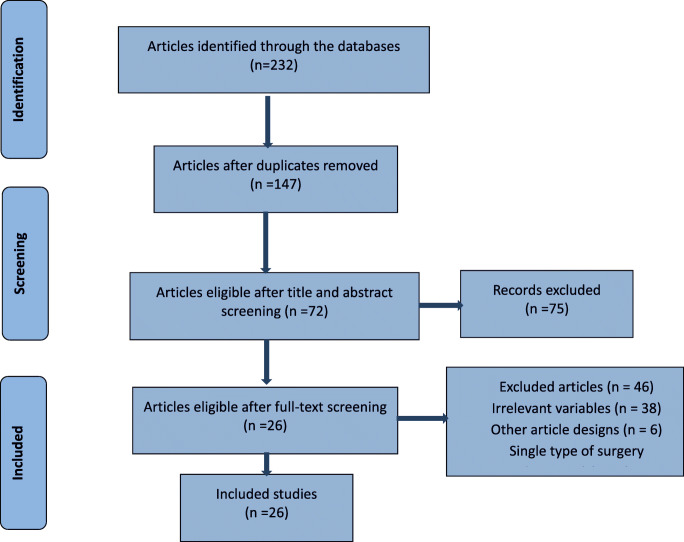
Preferred Reporting Items for Systematic Reviews and Meta-Analyses (PRISMA)

A total of 26 studies [8, 9, 17–40] examining 1771 patients were included in this meta-analysis (Fig. 1). Since the results of OAGB/MGB were classified and used as a revisional procedure in view of a primary procedure, some articles are quoted in more than one table (Tables 1, 2, and 3).

**Table 1:** Study characteristics with primary surgery of LSG included in meta-analysis

Author	Type of primary surgery	Number of patients	Interval to revision (month)	Pre-revision BMI (kg/m^2^)						Post-revision BMI (kg/m^2^)						Mean follow-up (year)
				At primary		Nadir		At revision		1 year		≤ 3 years		≤ 5 years		
				Mean	SD	Mean	SD	Mean	SD	Mean	SD	Mean	SD	Mean	SD	
Poublon2020 [31]	LSG	65	–	45.7	–	40.9	–	–	–	30.7	–	31.1	5.1	–	–	NA
Chevallier et al.,2015 [8]	LSG	NA	–	–	–	–	–	44.5	6.4	–	–	28.9	3.7	–	–	2.5
Lessing et al., 2017 [32]	LSG	27	105.6	–	–	–	–	42.2	8.3	–	–	–	–	–	–	4
Mora Oliver et al., 2019 [33]	LSG	26	70	–	–	–	–	–	–	–	–	–	–	–	–	1.6
Bruzzi et al., 2016 [24]	LSG	NA	34	–	–	–	–	45.5	7	33	4.5	30.5	4	32	5	5.5*
AlSabah et al., 2018 [22]	LSG	31	61	49		32.2	–	42.6		32.2	5.1	–	–	–	–	NA
Bhandari et al., 2019 [23]	LSG	32	–	44.04		34.22	–	38.53		34.33		37.14		–	–	NA
Moszkowicz et al.,2013 [18]	LSG	21	26.3	50.6	12.3	–	–	44	7.7	34.6	5.2	35.7	4.3			1.4
Poghosyan et al.,2019 [27]	LSG	72	28	49.1	8	–	–	43.6	7	34.6	5	33	9	34.7	9	NA
Debs et al., 2020 [26]	LSG	77	53.7	46.9		–	–	40.1		29.8		–	–	29.1	5	4.5
Jamal et al., 2020 [30]	LSG	56	–			–	–	41.9	7.9	30.5	9.4	–	–	–	–	1.5
Chiappetta 2019 [25]	LSG	34	38.5	56.5	8.8	–	–	45.7	8	36.6	6.3	–	–	–	–	NA
Musella et al., 2019 [19]	LSG	104	21.8	41.25	8.34	–	–	41.8	6.3	–	–	30.5	5.5	–	–	1.7
Lessing et al., 2020 [17]	LSG	4	142.8	46.7	5.9	–	–	42.8	7	31.3	5.2	–	–	–	–	1-year: 100% 2-year: 71.9%
Noun et al., 2018 [29]	LSG	7	–	45	4.8	35	5	42.9	6.5	28.5	4	–	–	–	–	NA

*Estimated mean using median and interquartile range (available at http://www.math.hkbu.edu.hk/~tongt/papers/median2mean.html)

**Table 2:** Study characteristics with primary surgery of LABG included in meta-analysis

Author	Type of primary surgery	Number of patients	Interval to revision (month)	Pre-revision BMI (kg/m^2^)						Post-revision BMI (kg/m^2^)						Mean follow-up (year)
				At primary		Nadir		At revision		1 year		≤ 3 years		≤ 5 years		
				Mean	SD	Mean	SD	Mean	SD	Mean	SD	Mean	SD	Mean	SD	
Noun et al., 2018 [29]	LAGB	10	–	45	4.8	35	5	42.9	6.5	28.5	4	–	–	–	–	NA
Noun et al., 2012 [40]	LAGB	77	–	41.25	8.34	–	–	–	–	–	–	–	–	–	–	1-year: 83.6% 1.5-year: 83% 3-years: 81% 4-years: 78% 5-years: 70
Lessing et al., 2020 [17]	LAGB	53	142.8	46.7	5.9	–	–	42.8	7	31.3	5.2	–	–	–	–	1-year: 100% 2-year: 71.9%
Poublon 2020 [31]	LAGB	120	–	45.7	–	40.9	–	–	–	30.7	–	31.1	5.1	–	–	NA
Musella et al., 2019 [19]	LAGB	196	21.8	41.25	8.34	–	–	41.8	6.3	–	–	30.5	5.5	–	–	1.7
Piazza et al., 2015 [20]	LAGB	47	28.5	–	–	–	–	43.4	4.2	34.1	3.77	–	–	–	–	NA
Chevallier et al.,2015 [8]	LAGB	41	–	–	–	–	–	44.5	6.4	–	–	28.9	3.7	–	–	2.6
Lessing et al., 2017 [32]	LAGB	71	105.6	–	–	–	–	42.2	8.3	–	–	–	–	–	–	4
Mora Oliver et al.,2019 [33]	LAGB	26	70	–	–	–	–	–	–	–	–	–	–	–	–	1.7
Chansaenroj et al.,2017 [34]	LAGB	26	–	39.9	10.5	–	–	39.3	8.9	27.4	5.2	26.8	4.8	–	–	5-year: 34.1% 10-years: 30.6%
Ghosh et al., 2017 [35]	LAGB	74	–	48.9	11.2	–	–	46	8.9	33.2	7.34	–	–	–	–	6-week: 97% 3-months: 85% 6-months: 69% 1-year: 46%
Noun et al., 2007 [36]	LAGB	16	36.3			–	–	39.5	10.4	30.6	4.77	–	–	–	–	0.6
Rutledge et al., 2006 [37]	LAGB	3	–	–	–	–	–	38.7	7			–	–	–	–	NA
Pujol Rafols et al., 2018 [28]	LAGB	191	–	44.3	6.8	–	–	39.8	6.9	–	–	–	–	30.3	5.4	2.8
Carbajo et al. 2017 [9]	LAGB	13	–	–	–	–	–	41.6	5.6	–	–	–	–	28.5	5.2	6-years: 87% 12-years: 70%
Bruzzi et al., 2016 [24]	LAGB	30	34	–	–	–	–	45.5	7	33	4.5	30.5	4	32	5	66.5

**Table 3:** Study characteristics with primary surgery of VBG included in meta-analysis

Author	Type of primary surgery	Number of patients	Interval to revision (month)	Pre-revision BMI (kg/m^2^)						Post-revision BMI (kg/m^2^)						Mean follow-up (year)
				At primary		Nadir		At revision		1 year		≤ 3 years		≤ 5 years		
				Mean	SD	Mean	SD	Mean	SD	Mean	SD	Mean	SD	Mean	SD	
Carbajo et al. 2017 [9]	VBG	14	–	–	–	–	–	41.6	5.6	–	–	–	–	28.5	5.2	7.5
Chevallier et al.,2015 [8]	VBG	NA	–	–	–	–	–	44.5	6.4	–	–	28.9	3.7	–	–	2.6
Bruzzi et al., 2016 [24]	VBG	30	34	–	–	–	–	45.5	7	33	4.5	30.5	4	32	5	5.8
Mora Oliver et al.,2019 [33]	VBG	36	70	–	–	–	–	–	–	–	–	–	–	–	–	1.7
Noun et al., 2007 [36]	VBG	17	36.3			–	–	39.5	10.4	30.6	4.8	–	–	–	–	0.8
Salama et al., 2016 [38]	VBG	39	–	–	–	–	–	39.7	8.2	30.2	5.4	–	–	–	–	NA
Wang et al., 2004 [39]	VBG	29	58.5	–	–	–	–	41.7		32.1		–	–	–	–	NA
Almalki et al., 2018 [21]	VBG	81	58.8	–	–	–	–	37.8	9.6	27.2	6.2	–	–	27.8	6.7	1-year: 60% 5-years: 37%

### Weight Regain Definition

The definitions provided for weight regain in the studies were different, but 33.3% of the studies relied on BMI ≥ 35 or EWL ≤ 50%. Also, EBMIL < 50% and EBMIL ≤ 25% were used for defining weight regain in 28.6% of the studies included in this review (Table 4).

**Table 4:** Type of weight regain definition used in included studies

Weight regain definition	Frequency	Rate %
BMI ≥ 35 or EWL ≤ 50%	7	33.3
BMI ≥ 35	2	9.5
EWL 2 years < 50% or > 25% EWL regain compared with minimal weight	1	4.8
EWL 2 years < 50%	1	4.8
EBMIL < 50%	3	14.3
EBMIL ≤ 25%	3	14.3
EWL < 50% 18 months after surgery	1	4.8
EWL < 50% at 18-month follow-up and EWL < 30% at any time	1	4.8
EWL < 50%, and BMI ≥ 50	1	4.8
EWL < 50% and/or TWL < 25% and/or BMI > 40 at 2 years follow-up	1	4.8

### Conversional OAGB/MGB Etiologies

**Fig. 2:**
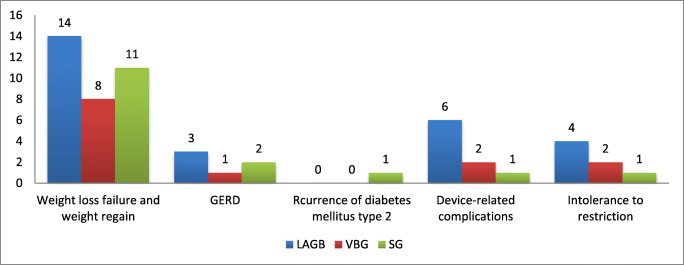
Frequency of reported etiologies for revisional OAGB/MGB in included studies

Weight regain and weight loss failure were the most frequent causes of revisional OAGB/MGB. Weight loss failure was reported as the most common etiology for revisional procedure. Other causes included abdominal pain/dyspepsia, port infection, device-related complications, or intolerance to restriction, such as band migration, slippage and port infection, recurrence of type-2 diabetes mellitus, gastroesophageal reflux disease (GERD), dysphagia, and esophageal disorders (Fig. 2).

### Biliopancreatic Limb Length

This review showed that the most common biliopancreatic limb length in OAGB/MGB was 200 cm (36% of the studies). The biliopancreatic limb length varied from 150 to 350 cm (Table 5).

**Table 5:** Biliopancreatic limb length used in OAGB/MGB surgeries in included studies

Biliopancreatic limb length	Frequency	Rate %
200 cm	9	36
180–200 cm	1	4
180–240 cm	1	4
150 cm and 200 cm	2	8
180 cm	2	8
150–200 cm	1	4
175 -200 cm	1	4
250 cm	1	4
150 cm	2	8
175 cm	1	4
150 cm (and increased by 10 cm for each BMI point above 40)	1	4
150–250 cm	1	4
150–300 cm	1	4
250–350 cm tailored	1	4

### Weight Loss Outcomes at 1-, 3-, and 5-Year Follow-Ups

**Fig. 3:**
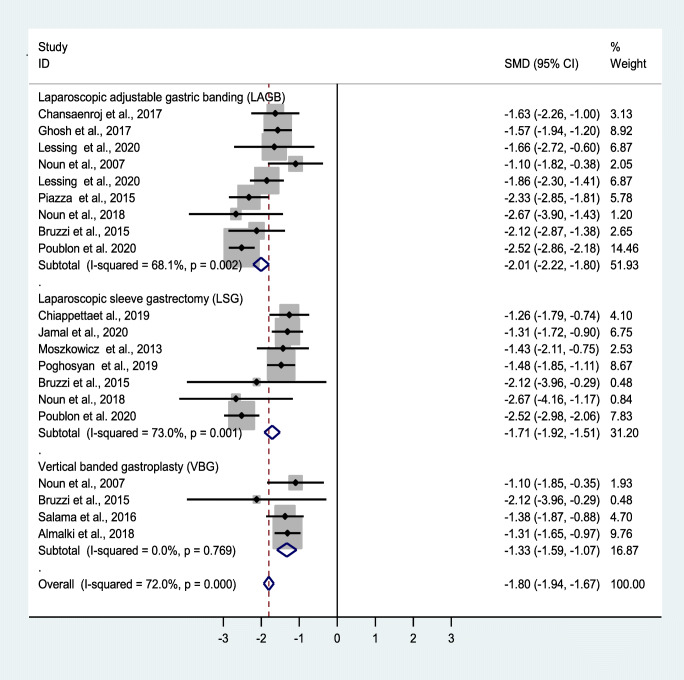
Forest plot showing the effect of OAGB/MGB on BMI after 1-year follow-up

**Fig. 4:**
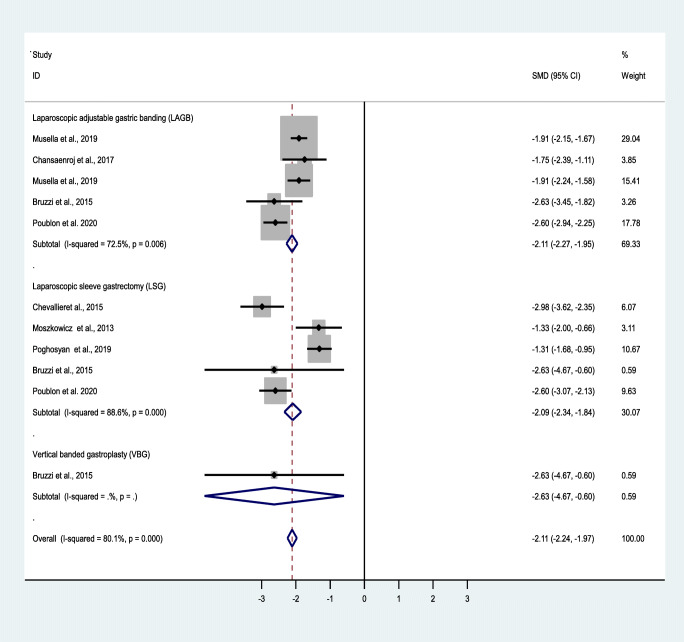
Forest plot showing the effect of OAGB/MGB on BMI after 3-year follow-up

**Fig. 5:**
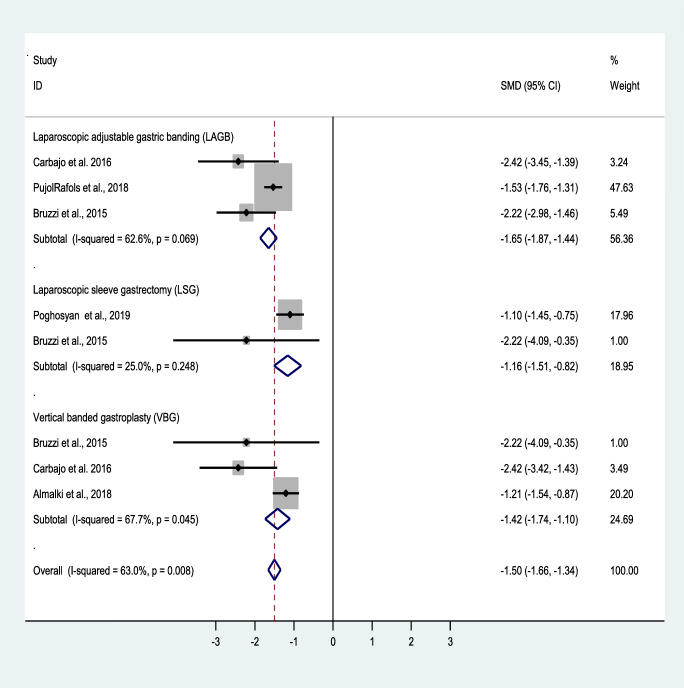
Forest plot showing the effect of OAGB/MGB on BMI after 5-year follow-up

The mean initial BMI was 45.70 kg/m^2^, which decreased to 31.5, 31.4, and 30.5 kg/m^2^ at 1-, 3-, and 5-year follow-ups, respectively (Table 6). The forest plots depict the effects of OAGB/MGB on BMI after 1, 3, and 5 years (Figs. 2, 3, 4, and 5).

**Table 6:** Mean and standard deviation of reported BMI at revision time and follow-up

Variable	Number of patients	Mean ± Std. deviation
At revision BMI (kg/m^2^)	1584	42.3 **±** 2.2
Post-revision BMI ≤ 1 year (kg/m^2^)	761	31.6 **±** 2.5
Post-revision BMI ≤ 3 years (kg/m^2^)	658	31.4 **±** 3.1
Post-revision BMI ≤ 5 years (kg/m^2^)	505	30.6 **±** 2.3

A random effect model was also used to measure the effect of the standardized mean difference (SMD). Based on the results of the included studies, OAGB/MBG has been effective in accomplishing weight loss. Nonetheless, at 1-, 3-, and 5-year follow-ups, BMI decreased by an SMD of − 1.8, − 2.1, and − 1.5, showing the acceptable and durable effects of OAGB/MGB. At the 1-year follow-up, OAGB/MGB following LAGB was most effective in decreasing BMI, with an SMD of − 2.01. Also, OAGB/MGB following LSG (SMD = − 1.7) showed a better effectiveness compared to VBG (SMD = − 1.3). In the 3-year follow-up, OAGB/MGB after LAGB was most effective in decreasing BMI with an SMD of − 2.1. In the 5-year follow-up, OAGB/MGB following LAGB was most effective in decreasing BMI, with an SMD of − 1.7. In this follow-up, conversional OAGB/MGB after VBG (SMD = − 1.4) played an effective role in BMI reduction. The effect of OABG on BMI after a 1-year follow-up with the presence of remnant resection showed no significant difference between the two states (Table 11).

### Comorbidity and Remission

The comorbidities included in this meta-analysis were T2DM [3], hypertension (HTN), obstructive sleep apnea (OSA), and dyslipidemia (DL) (Tables 7 and 8).

**Table 7:** Pre-revision, at revision, and post-revision comorbidities after OAGB/MGB

Author/year	Number of patients	Post-revision comorbidities remission											
		1 year				≤ 3 years				≤ 5 years			
		HTN	T2DM	OSA	DL	HTN	T2DM	OSA	DL	HTN	T2DM	OSA	DL
AlSabah 2018 [22]	31	50	–	–	–	–	–	–	–	–	–	–	–
Bhandari 2019 [23]	32	81.8	88.8	–	–	81.8	88.8	–	–	81.8	71.4	–	–
Chiappetta 2019 [25]	34	100	66.7	80	61.5	–	–	–	–	–	–	–	–
Debs 2020 [26]	77	–	–	–	–	–	–	–	–	65	62	82	–
Jamal 2020 [30]	56	42	40	–	–	–	–	–	–	–	–	–	–
Poghosyan 2019 [27]	72	–	–	–	–	21	75	70	–	–	–	–	–
Musella 2019 [19]	300	–	–	–	–	45	12	–	11	–	–	–	–
Bruzzi 2016 [24]	30	–	–	–	–	–	–	–		58	85	–	75
Chevallier 2015 [8]	177	–	–	–	–	52.1	85.7	50	80.6	–	–	–	–
Carbajo 2017 [9]	27	–	–	–	–	–	–	–	–	94	94	90	96
Poublon 2020 [31]	185	–	–	–	–	–	96.9	87.5	80	–	–	–	–

**Table 8:** Remission of HTN, T2DM, OSA, and DL following OAGB/MGB surgery at 1-, 3-, and 5-year follow-up

Post-revision remission rate range	Minimum (%)	Maximum (%)	Mean ± Std. deviation
HTN at 1-year follow-up	42	100	68.5 ± 27.2
T2DM1 at 1-year follow-up	40	88.8	65.2 ± 24.4
OSA at 1-year follow-up	80	80.0	80 ± 0
DL at 1-year follow-up	61	61.5	61.5 ± 0
HTN at 3-year follow-up	21	81.8	49.9 ± 25
T2DM at 3-year follow-up	12	88.8	65.4 ± 36.1
OSA at 3-year follow-up	50	70.0	60 ± 14.1
DL at 3-year follow-up	11	80.6	45.8 ± 49.2
HTN at 5-year follow-up	58	94.0	74.7 ± 16.3
T2DM at 5-year follow-up	62	94.0	78.1 ± 14.2
OSA at 5-year follow-up	82	90.0	86 ± 5.7
DL at 5-year follow-up	75	96.00	85.5 ± 14.8

### Diabetes Outcomes

Remission of T2DM following OAGB/MGB surgery at 1-, 3-, and 5-year follow-up was 65.2 ± 24.4, 65.4 ± 36.1, and 78.1 ± 14.2, respectively. The remission range of T2DM at the 1-, 3-, and 5-year follow-ups was 40–88.8%, 12–88.8%, and 62–94%, respectively (Table 8).

### HTN Outcomes

Remission of HTN following OAGB/MGB surgery at 1-, 3-, and 5-year follow-ups was 68.4 ± 27.1, 49.9 ± 25, and 74.7 ± 16.2%, respectively. The remission range for HTN at 1-, 3-, and 5-year follow-ups was 42–100%, 21–81.8%, and 58–9%, respectively (Table 8).

### Dyslipidemia Outcomes

Remission of DL following OAGB/MGB surgery at 1-, 3-, and 5-year follow-ups was 61.5 ± 0, 45.8 ± 49.2, and 85.50 ± 14.8, respectively. The remission range for DL at 1-, 3-, and 5-year follow-ups was 61–61.5%, 11–80.6%, and 75–96%, respectively (Table 8).

### OSA Outcomes

Remission of OSA following OAGB/MGB surgery at 1-, 3-, and 5-year follow-ups was 80.00 ± 0, 60.00 ± 14.1, and 86.00 ± 5.7, respectively. The remission range for OSA at 1-, 3-, and 5-year follow-ups was 80–80%, 50–70%, and 82–90%, respectively (Table 8).

### GERD Outcomes

GERD following OABG surgery was also investigated. The results showed that 81.7% of the patients with GERD improved or had remission following OAGB/MGB (Table 9).

**Table 9:** Rate of preoperative and postoperative GERD in revisional OAGB/MGB surgery

Author/year	Pre-revisionGERD (%)	Post-revision GERD (%)	Remission (%)	Remission mean ± SD
Noun 2007 [36]	18.2	0	100	81.8 ± 29.7
Piazza 2015 [20]	8.3	0	100	
Chiappetta 2019 [25]	14.7	11.8	19.7	
Almalki 2018 [21]	18.5	–	–	
Musella 2019 [19]	4.6	2	56.5	
Lessing 2020 [17]	29.8	0	100	
Bruzzi 2016 [24]	13	–	–	
Lessing 2017 [32]	9.1	0	100	
Carbajo 2017 [9]	29.6	0	100	
Poublon 2020 [31]	10.2	2.2	78	

The results showed that 7.4% of the patients developed de novo GERD following OABG.

### Major Complications

The major complications reported in the studies were extracted, and leakage proved to be the most common problem after revisional OAGB/MGB (0.016). The other complications included hematoma and abscess, GIB, reoperation, strangulated hernia at the trocar port, late incisional hernia, colonic necrosis, bowel obstruction, respiratory failure, anastomotic stricture, hypoalbuminemia, intractable bile reflux, small bowel ileus, pneumonia, GJ stoma fistula, hematemesis, port site infection, and ulceration (Table 10).

**Table 10:** Major complication rate reported in included studies

Complication	Total complication in 1771 patients	Rate %
Leakage	29	1.6
Bleeding	23	1.2
Hematoma and abscess	12	0.6
GIB	2	0.1
Hypoalbuminaemia	4	0.2
Reoperation	2	0.1
Strangulated hernia at trocar port	2	0.1
Late incisional hernia	4	0.2
Colonic necrosis	1	0.06
Bowel obstruction	3	0.1
Respiratory failure	1	0.06
Stricture	4	0.2
Intractable bile reflux	2	0.1
Small bowel ileus	2	0.1
Pneumonia	1	0.06
GJ stoma fistula	1	0.06
Hematemesis	1	0.06
Port site infection	1	0.06
Ulceration	4	0.2

### Publication Bias

The results of the analysis also showed that bias publication did not have an influence on the creation of negative results, which is shown as symmetry in the funnel plot. Meanwhile, no evidence of publication bias was detected using Egger’s test (Egger’s test *t* = − 2.03, *P* = 0.06, 95% CI − 3.4 to 0.08).

## Discussion

As reported in previously published studies [41, 42], weight loss failure and weight regain may occur in the long term following restrictive procedures. This review study revealed unsuccessful weight loss as the main reason for conversion to OAGB/MGB. All the 57 patients reported by Lessing et al. were converted to OAGB/MGB due to weight regain after gastric banding either by the 1-stage or 2-stage approach [17]. Also, all the reported revisional OAGB/MGBs in Moszkowicz’ series were due to weight regain [18]. In the study by Musella et al., 77% of the restrictive operations (sleeve gastrectomy or banding) were converted due to weight loss failure, and only 23% of the revisions were due to surgical complications (dysphagia, disconnection of tube from the port, port infection and slippage, deterioration of preoperative gastroesophageal reflux (GERD), or de novo GERD) [19]. Band-related complications (slippage, migration, pouch dilation) (43%), esophageal disorders (31%), weight loss failure/persistence of comorbidities (15%), and food intolerance/patient request (11%) were the indications for revisional OAGB/MGB in the other retrieved papers [20].

While insufficient weight loss was the principal reason for conversion, BPL length was found to represent a crucial technical point regarding revisional surgery. Nonetheless, in this review study, BPL varied from 150 to 350 cm, as the optimal limb length in primary and revisional OAGB/MGB is still a matter for debate. The results of this research showed that the most common BPL length in OAGB/MGB was 200 cm (36% of the studies). Meanwhile, some studies used 150-cm BPL lengths (and increased them by 10 cm for each BMI point above 40) [9, 21–26, 43, 44]. Poghosyan et al. found that weight loss and its outcomes are comparable between the 150-cm and 200-cm BPL [27]. Tovar et al. found that the ideal range was established between 0.40 and 0.43 for the CL/TBL ratio, and 200 and 220 cm for the CL length. Among these ranges, there were no cases of protein or calorie malnutrition [45]; therefore, it is better to measure the entire small intestine and use a maximum one-third of it for GJ to prevent malnutrition.

Even though further studies are needed to identify the optimal length for primary and revisional OAGB/MGB, the present research demonstrated that, regardless of BPL, conversion from restrictive interventions has resulted in satisfactory and durable outcomes in series with 5-year follow-ups [9, 21, 24, 26–28]. According to pre and post-revisional BMI between groups, BMI loss was more significant after LAGB compared to OAGB/MGB following LSG or VBG, perhaps due to the lesser weight loss after LAGB as an initial bariatric surgical procedure in comparison with LSG and VBG. This finding may suggest that LAGB can have better post-revisional weight loss outcomes than LSG before gastric bypass [41]. This review of literature also showed that simultaneous gastric remnant resection, which was performed in some of the studies [21, 23, 25, 26, 29], has no significant effects on weight loss, but, in our opinion, it can only increase the risk for postoperative complications and hamper reversal surgery (Table 11).

**Table 11:** Effect of gastric remnant resection on BMI following revisional OAGB/MGB at 1 year of follow-up

Variable	Remnant resection (*n* = 319)	Mean	*t*	*P* value
Post-revision BMI ≤ 1 year (kg/m^2^(	Yes	31.6 ± 3.7	− 0.19	0.86
	No	31.9 ± 2.2		
Post-revision BMI ≤ 3 years (kg/m^2^)	Yes	37.1 ± 0	1.85	0.12
	No	30.9 ± 3.1		
Post-revision BMI ≤ 5 years (kg/m^2^)	Yes	28.5 ± 0.9	− 2.25	0.11
	No	32.3 ± 2.2		

Regarding the effects of conversion on T2DM, the present data demonstrated a range of remission up to 65–78% during the first 5 years. In Bruzzi’s series, the remission rate for diabetic patients after revisional OAGB/MGB was 85% [24], while another study showed a 100% remission of T2DM after revisional OAGB/MGB compared to 60% remission after revisional RYGB [25]. Debs et al. also reported that ten out of 13 patients had remission or improvement of T2DM after revisional OAGB/MGB [26]. Musella et al. reported a 75% and 50% remission of T2DM after failed primary sleeve gastrectomy and adjustable banding, respectively, as restrictive operations converted to OAGB/MGB [19].

Revisional OAGB/MGB operation also causes improvements in other comorbidities, such as HTN, with remission rates of 58–94% 5 years following the redo surgery. Chiappetta et al. concluded that a 1-year follow-up leads to a greater metabolic improvement after OAGB/MGB following failed LSG compared to RYGB [25]. In the study by Debs et al., HTN was resolved or improved in 19 out of the 23 patients (82%) after 55 months [26]. Similarly, in another study by Jamal et al., out of 19 HTN patients, all had normalized blood pressure 1 year after OAGB/MGB, and no longer need to take any antihypertensive medications or at least decreased their medication intake [30]. Conversely, Musella et al. reported a lower rate (40%) of remission from HTN in their series [19].

In addition to T2DM remission, this analysis found an improvement in the lipid profile. Some articles did not report the DL outcomes after OAGB/MGB as a revisional operation [17, 21, 23, 26, 30]. Nevertheless, a 75% remission rate of DL was reported by Bruzzi et al. [24]. Chiappetta et al. also showed a 25% and 61.5% improvement in DL after revisional RYGB and OAGB/MGB, respectively [25]. Another study revealed a 56% remission of dyslipidemia after revision of failed sleeve gastrectomy or adjustable band to OAGB/MGB [19]. Lipid profile change after primary OAGB/MGB was also reported by Milone et al., and more than 50% of the patients had a normal lipid profile after primary OAGB/MGB [46].

Furthermore, an OAGB/MGB operation also caused improvements in OSA, with average remission rates of 86% in the 5-year follow-up. In the study by Debs et al., HTN was resolved in 27 out of the 33 patients (82%) after 55 months [26]. In the study by Bruzzi et al., no significant differences were found in the remission rates of any obesity-related comorbidity such as OSA, which had occurred in the revisional and primary groups. These authors reported a 50% remission rate for OSA in both groups [24]. Similarly, Piazza et al. found a 66% remission only 6 months after OAGB/MGB following failed adjustable GB [20].

Although there is a clear difference between acid reflux and bile reflux [47], the risk of GERD and/or bile reflux after primary and revisional OAGB/MGB is currently debated in literature [48, 49]. Present meta-analysis showed that conversional OAGB/MGB can lead to GERD improvement in approximately 82% of patients. Only three studies reported de novo GERD and bile reflux (BR) in the patients who had no GERD symptoms before conversional OAGB/MGB [10, 26, 27]. A defective surgical technique could be the reason for reflux after conversional OAGB/MGB, especially due to a short gastric pouch [10, 50, 51] or the presence of undetected and non-repaired hiatal hernia [52].

Redo surgery is often burdened by a higher rate of postoperative complications due to the more complicated surgery procedure; however, even though revisional OAGB/MGB needs skilled and expert surgeons, the intervention itself appears to be more feasible than classic RYGB or other malabsorptive operations. The present review showed that complications such as port site infection, abscess, trocar site hernia, incisional hernia, colonic necrosis, bowel obstruction and ileus, pneumonia, and anastomotic stricture were within the range of primary OAGB/MGB. The first most common complication was leakage, with a rate of 1.6%, which is slightly higher than for the primary procedure, and the second most common complication was bleeding, with a rate of 1.2%, which was comparable to that for primary OAGB/MGB [12, 14, 53–55]. The rate of hypoalbuminemia was only 0.2% in 1771 patients although most studies report a BPL of 200 cm. It may be under-reported as a major complication in some studies or lesser effect on albumin as a revisional procedure in patient who had an initial bariatric procedure.

### Strength and Limitations

It must be considered no RCTs on OAGB/MGB as revisional surgery following restrictive procedures have been published so far. Although a similar paper has recently been published [14], this study is the first systematic review in which a meta-analysis has been developed. Also, conversion surgery data from VBG to OAGB/MGB have been evaluated. Main limitation is surely represented by the retrospective setting of all studies we considered. Again, although recalled in Table 4, it must be noted that definition for weight regain is not univocal in many of the papers we retrieved.

## Conclusion

OAGB/MGB as a revisional procedure after failed restrictive bariatric surgery is feasible and effective. Regardless of the BPL length, conversion to OAGB/MGB induces further weight loss after LSG, VGB, and especially LAGB. The rate of remission of classic obesity-related diseases after this procedure is satisfactory, and its postoperative complications are comparable to those of primary OAGB/MGB.

## Electronic Supplementary Material

ESM 1 (DOC 62 kb).
